# Assessment of adherence to treatment recommendations among patients with heart failure: a cross-sectional study

**DOI:** 10.1186/s12872-024-04001-y

**Published:** 2024-07-04

**Authors:** Aleksandra Kukulska, Elżbieta Garwacka-Czachor

**Affiliations:** Medical Institute, State University of Applied Sciences in Głogów, Piotra Skargi 5 Street, Głogów, 67- 200 Poland

**Keywords:** Heart failure, Cardiovascular diseases, Medication adherence, ACDS

## Abstract

**Background:**

Heart failure (HF) is a chronic condition characterized by significant impairment of the cardiovascular system, leading to a decline in health-related quality of life, recurrent hospitalizations, and increased mortality risk. It poses a substantial challenge for modern medicine, particularly when patients fail to adhere to therapeutic recommendations. The primary aim of this study was to evaluate the level of adherence to therapeutic guidelines among patients with HF and identify factors influencing adherence levels.

**Methods:**

The study comprised 105 HF patients admitted to the cardiology department. A diagnostic survey approach was utilized, employing the Adherence in Chronic Diseases Scale (ACDS) along with a self-developed questionnaire.

**Results:**

The findings revealed that 39.05% of participants exhibited a moderate level of adherence to therapeutic recommendations, while 34.29% reported high adherence and 26.67% displayed low adherence. Most of the patients (*n* = 66) had a rather good level of knowledge. Factors such as higher education (*p* < 0.001), engagement in mental work (*p* = 0.001), favorable socioeconomic status (*p* < 0.001), being in a stable relationship (*p* < 0.001), and residing with family (*p* < 0.001) were associated with increased adherence levels. The multivariable linear regression model indicated significant (*p* < 0.05) independent predictors that positively influenced the ACDS score, including being in a relationship, widowhood, and average or poor financial situation. Conversely, factors such as obesity and respiratory diseases were associated with a decrease in the ACDS score (*p* < 0.05).

**Conclusions:**

This study underscores the moderate adherence level to therapeutic recommendations among HF patients. Sociodemographic factors including education level, relationship status, occupation, financial stability, and living arrangements significantly impact adherence. Conversely, patients with obesity, respiratory conditions, or frequent HF-related hospitalizations demonstrate lower adherence. Patient education emerges as a pivotal factor influencing adherence. Tailored interventions targeting these factors could enhance adherence and optimize HF management outcomes.

## Background

Heart failure (HF) is a chronic condition that often leads to significant impairment of the cardiovascular system [1]. Epidemiological data suggest that in developed countries, approximately 2% of the adult population suffers from HF [2]. In the United States alone, around 6.7 million individuals aged 20 and older are affected by HF, with projections indicating a rise to 8.5 million by 2030. Pre-HF conditions affect about 24–34% of adults in the US [3]. The ATLAS study conducted by the Heart Failure Association (HFA) in 2019 revealed varying HF prevalence rates across Europe, ranging from ≤ 12 per 1000 individuals in Spain and Greece to over > 30 in Lithuania and Germany [4]. In Asia, HF prevalence ranges from 1.3 to 6.7% [5]. Anticipated increases in HF prevalence globally are attributed to factors like population aging and advancements in coronary heart disease treatment, including evidence-based therapies for HF, especially HF with reduced ejection fraction (HFrEF) [5].

In Poland, the number of HF patients in 2019 was 1.02 million, with a positive trend seen in the decline of de novo HF diagnoses, registering 127,000 patients in 2019, a 26% reduction from 2014 [6]. HF is a leading cause of hospitalization for individuals aged 65 and older in Poland, with nearly 180,000 admissions annually and more than half requiring rehospitalization. Additionally, one in four patients undergoes inpatient treatment within 30 days of discharge [7, 8]. Despite advancements in cardiovascular disease treatment, the annual mortality rate from HF remains high, with approximately 140,000 HF-related deaths reported in Poland in 2018, representing over 30% of all deaths during that period [8].

Treatment for HF patients involves lifestyle changes and pharmacological therapy. Adherence to therapeutic recommendations is essential, encompassing medication adherence, maintaining a balanced diet, regular physical activity, weight monitoring, substance avoidance, and specialist check-ups [9]. Non-adherence to pharmacotherapy and healthy lifestyle recommendations poses various risks [10].

So far, researchers have identified numerous factors influencing adherence to therapeutic recommendations in HF patients. The World Health Organization classifies these factors into five main groups: sociodemographic, clinical, healthcare system-related, treatment-related, and patient-related [11]. Studies involving HF-diagnosed patients confirm the impact of gender, education level, marital status, age, income, therapy, side effects, treatment costs, and comorbidities on adherence [10, 12, 13]. Additionally, lack of symptoms or mild intensity, substance use, depression, lack of support, and low disease knowledge are significant indicators of both intentional and unintentional non-adherence [14–16].

Consequences include reduced treatment effectiveness, frequent hospitalizations, complications, drug resistance, and decreased health-related quality of life (HRQoL), with premature death being the most severe outcome, albeit preventable. Non-adherence also disrupts the treatment process, necessitating specialist visits, additional tests, new medications, and sometimes hospitalization [17]. This results in lost time, financial costs, and chronic health impairment, leading to work disability, increased sick leave costs, and reduced employer productivity. The healthcare system also bears the burden, with non-adherence costs in Poland totaling approximately 1.4 billion EUR annually [18, 19].

In Poland, the healthcare system operates under a mix of public and private services, with the majority of the population relying on the public sector [20]. Economic disparities and regional differences can affect access to care and resources for chronic disease management. Additionally, cultural attitudes towards healthcare and adherence may vary, impacting the implementation of therapeutic recommendations [21, 22]. Highlighting these specific factors within the Polish context can provide valuable insights into the challenges and facilitators of adherence in HF management [23], which may also be relevant to other countries with similar healthcare dynamics.

While existing evidence emphasizes medication adherence’s importance in HF management, further research is crucial to explore factors influencing adherence, develop innovative interventions, and assess long-term clinical outcomes. Continued research is essential for refining strategies to optimize medication adherence, leading to improved health outcomes for HF patients. Therefore, this study aims to assess adherence levels and determine influencing factors among HF patients.

## Methods

### Study participants

The study was conducted at the Provincial Integrated Hospital in Leszno, Poland, involving 105 patients hospitalized in the cardiology department. The research took place from June to December 2022, with patients having the option to withdraw at any stage, and incorrectly filled surveys were excluded. Approval was obtained from the local Bioethics Committee of the Medical Institute at the State University of Applied Sciences in Głogów, Poland (no. 75/2022), adhering to the principles of the Helsinki Declaration and Good Clinical Practice, and following the STROBE guidelines for comprehensive and transparent reporting of observational studies.

Inclusion criteria comprised obtaining informed consent, clinical diagnosis of HF, stable medical condition, adult age, receipt of HF-specific treatment, varying duration of HF diagnosis, language proficiency, and absence of severe comorbidities, ensuring a focused and representative sample. Exclusion criteria included unwillingness to participate, cognitive impairment hindering questionnaire completion, acute exacerbation of HF, severe comorbidities (e.g., terminal illness, severe psychiatric disorders), recent major surgery, and inability to understand or communicate in the questionnaire language, aimed at excluding potential confounders or data quality compromise.

### Research tools

The research methodology utilized in the study employed the diagnostic survey method with a questionnaire technique. The tools utilized are described below. The questionnaire, designed by the authors, consists of 21 questions focusing on sociodemographic data, including gender, age, place of residence, education, marital status, and occupational activity. The second part of the survey addresses selected medical aspects and health behaviors such as smoking, alcohol consumption, adherence to dietary recommendations, and engagement in physical activity. The respondents’ level of knowledge was assessed based on their answers to question number 17. Each domain was evaluated on a scale ranging from very poor (0 points) to very good (4 points).

The Adherence in Chronic Diseases Scale (ACDS) comprises 7 questions with proposed response sets. These questions address behaviors directly influencing adherence, as well as situations and beliefs impacting compliance with recommendations. The scale is designed for research among adults with chronic diseases, reflecting the actual implementation of the therapeutic plan and illustrating the mechanisms influencing participants’ adherence [24].

### Statistical analysis

The analysis of quantitative variables (i.e., expressed in numbers) involved calculating the mean, standard deviation, median, and quartiles. Group comparisons were performed using the chi-square test (with Yates’ correction for 2 × 2 tables) or the exact Fisher test in cases of low expected counts. Additionally, an analysis of quantitative variables (i.e., expressed in numbers) was conducted by calculating the mean, standard deviation, median, and quartiles. Comparison of these values between groups was performed using Mann-Whitney test (for two groups) or Kruskal-Wallis test (for more than two groups). Dunn’s test was used as post-hoc procedure after Kruskal-Wallis test. Also, both univariate and multivariable regression analyses were conducted to assess the impact of sociodemographic and clinical variables on treatment adherence. A significance level of 0.05 was adopted, with p-values below 0.05 considered indicative of significant dependencies. The statistical analysis was performed using R software, version 4.2.2 [25].

## Results

The sociodemographic characteristics of the studied patients are presented in Table 1. The study comprised 105 individuals with HF, including 39 women (37.14%) and 66 men (62.86%). The age distribution was as follows: 6 individuals (5.71%) were aged 20–40 years, 11 patients (10.48%) were aged 41–50, 16 respondents (15.24%) were aged 51–60, 41 patients (39.05%) were aged 61–70, 21 individuals were aged 71–80, and 10 participants (9.52%) were aged 80 years and above. Among the surveyed, 60 (57.14%) individuals resided in urban areas, and 45 (42.86%) came from rural areas. Education levels varied, with 8 respondents (7.62%) having basic or middle school education, 39 (37.14%) having vocational education, 44 (41.90%) having secondary education, 4 (3.81%) holding a bachelor’s degree, and 10 (9.52%) having a master’s degree. Regarding marital status, 6 (5.71%) individuals were single, 55 (52.38%) were in a formal relationship, 4 (3.81%) were in a domestic partnership, 31 (29.52%) were widowed, and 9 (8.57%) were divorced. Employment status varied, with 4 (3.81%) unemployed individuals, 18 (17.14%) engaged in mental work, 17 (16.19%) engaged in physical work, and 66 (62.86%) retirees. In terms of financial situation, 59 (56.19%) patients assessed it as good, 28 (26.67%) as average, 16 (15.24%) as very good, and 2 (1.90%) as poor. Living arrangements showed that 83 (79.05%) patients lived with their families, while 22 (20.95%) lived alone.

**Table 1:** Sociodemographic characteristics of the studied patients

Variable		*n*	%
Sex	Female	39	37.14%
	Male	66	62.86%
Age	20–40 years	6	5.71%
	41–50 years	11	10.48%
	51–60 years	16	15.24%
	61–70 years	41	39.05%
	71–80 years	21	20.00%
	80 years and over	10	9.52%
Place of residence	City	60	57.14%
	Village	45	42.86%
Education	Elementary or gymnasium	8	7.62%
	Vocational	39	37.14%
	Secondary school	44	41.90%
	Bachelor’s degree	4	3.81%
	Master’s degree	10	9.52%
Marital status	Single	6	5.71%
	Formal relationship	55	52.38%
	Partnership relationship	4	3.81%
	Widowed	31	29.52%
	Divorced	9	8.57%
Professional activity	Mental work	18	17.14%
	Physical work	17	16.19%
	Unemployment	4	3.81%
	Retirement/pension	66	62.86%
Material situation	Very good	16	15.24%
	Good	59	56.19%
	Average	28	26.67%
	Bad	2	1.90%
Inhabitation	Alone	22	20.95%
	With family	83	79.05%

The clinical characteristics of the studied patients are detailed in Table 2. The majority of respondents had been ill for 1 to 4 years (51.43%), followed by 5 to 10 years (33.33%), less than a year (13.33%), and above 11 years (1.90%). The most prevalent chronic diseases among the respondents were hypertension (60%), diabetes (40.95%), and heart rhythm disorders (30.48%). Regarding hospitalizations, 44.76% of participants did not stay in the hospital in the last year, 43.81% were hospitalized 1–2 times, 10.48% had 3–5 hospitalizations, and 0.95% had more than 5 hospitalizations. Smoking habits varied, with 76.19% of respondents not smoking cigarettes at all, while among smokers, the average duration of the habit was 21.83 years (SD = 9.31). Alcohol consumption was reported by 45.71% of respondents, with 54.29% abstaining. Dietary adherence levels varied, with 42.86% reporting a rather high level of adherence and 3.81% admitting to not adhering at all. Physical activity levels showed that 32.38% of respondents were active 1–2 times a week, while 4.76% did not engage in sports at all.

**Table 2:** Clinical characteristics of the studied patients

Variable		*n*	%
Duration of disease	Less than 1 year	14	13.33%
	1–4 years	54	51.43%
	5–10 years	35	33.33%
	Over 11 years	2	1.9%
Comorbidities	No	4	3.81%
	Diabetes	43	40.95%
	Atherosclerosis	19	18.1%
	Obesity	24	22.86%
	Hypertension	63	60.0%
	Respiratory diseases	22	20.95%
	Cardiac rhythm disorders	32	30.48%
	Other **	24	22.86%
Hospitalizations for HF exacerbations in the last year	Lack of hospitalizations	47	44.76%
	1–2 hospitalizations	46	43.81%
	3–5 hospitalizations	11	10.48%
	More than 5 hospitalizations	1	0.95%
Current cigarette smoking	At all	80	76.19%
	Occasionally	3	2.86%
	1–5 pieces a day	7	6.67%
	5–10 pieces a day	9	8.57%
	More than 10 pieces a day	6	5.71%
Use of electronic cigarettes	Yes	1	0.95%
	No	104	99.05%
Duration of addiction [years]	10	4	16.0%
	13	1	4.0%
	15	3	12.0%
	16	1	4.0%
	20	4	16.0%
	25	3	12.0%
	30	4	16.0%
	33	1	4.0%
	40	2	8.0%
	No answer	2	8.0%
Current alcohol consumption	At all	57	54.29%
	Occasionally	37	35.24%
	Once a week	7	6.67%
	Several times a week	3	2.86%
	Every day	1	0.95%
Physical activity	Every day	5	4.76%
	2–3 times a week	13	12.38%
	1–2 times a week	34	32.38%
	Several times a month	31	29.52%
	1–2 times a month	9	8.57%
	Less than once a month	8	7.62%
	Not at all	5	4.76%

*Note * * Percentages do not add up to 100, as this was a multiple-choice question; ** Primarily thyroid and kidney diseases

The level of adherence to therapeutic recommendations among the surveyed patients varied (Table 3). Out of 105 survey participants, 41 individuals (39.05%) exhibited moderate adherence, 36 respondents (34.29%) demonstrated high adherence, and 28 respondents (26.67%) showed low adherence.

**Table 3:** Level of adherence to therapeutic recommendations by surveyed patients

ACDS [score]	Interpretation	*n*	%
0–20	Low adherence	28	26.67%
21–26	Medium adherence	41	39.05%
27–28	High adherence	36	34.29%

*Abbreviations * ACDS – Adherence in Chronic Diseases Scale

Table 4 presents the results of a univariate analysis of the impact of selected sociodemographic variables on adherence to therapeutic recommendations by the surveyed patients. Age did not have a statistically significant impact on adherence to therapeutic recommendations (*p* > 0.05). Similarly, the place of residence of the respondents did not significantly influence adherence (*p* > 0.05). However, education showed statistical significance (*p* < 0.05), with individuals having higher education demonstrating higher adherence compared to those with intermediate, primary, junior high, or vocational education. Marital status also proved to be statistically significant (*p* < 0.05), with adherence being notably higher in individuals in a relationship compared to other groups. Occupational activity significantly impacted adherence (*p* < 0.05), with individuals engaged in mental work showing higher adherence than those in other groups. Financial situation was statistically significant (*p* < 0.05), with adherence being higher in respondents with better financial situations. Moreover, living arrangements (alone or with family) were statistically significant (*p* < 0.05), with adherence being higher among individuals living with family. Gender, however, was not statistically significant (*p* > 0.05) and did not influence adherence to therapeutic recommendations.

**Table 4:** The results of the univariate analysis of the impact of sociodemographic variables on the level of adherence to therapeutic recommendations by the surveyed patients

Variable		N	ACDS [score]							p
			Mean	SD	Median	Min	Max	Q1	Q3	
Sex	Female	39	23.74	4.52	26	10	28	21	27	*p* = 0.104*
	Male	66	21.80	6.23	23	3	28	19	27	
Age	20–40 years	6	18.50	12.13	25	3	28	8	27.00	*p* = 0.12*
	41–50 years	11	23.91	4.46	26	15	27	23	27.00	
	51–60 years	16	24.00	4.27	26	15	28	21	27.00	
	61–70 years	41	22.88	5.80	25	3	28	21	27.00	
	71–80 years	21	22.79	4.35	22	14	28	20	27.00	
	80 years and over	10	19.00	4.40	19	13	26	15	21.75	
Place of residence	City	60	23.12	5.58	26	3	28	21	27	*p* = 0.138**
	Village	45	21.73	5.85	23	3	28	19	27	
Education	Elementary or gymnasium - A	8	17.96	7.04	19	3	25.67	17.00	21.75	*p* < 0.001*** D > C > B,A
	Vocational - B	39	21.13	5.67	22	3	28.00	18.00	26.00	
	Secondary school - C	44	23.57	4.94	26	3	28.00	21.75	27.00	
	Higher education - D	14	25.71	5.14	27	8	28.00	27.00	27.00	
Marital status	Single - A	6	15.67	10.65	18.5	3	28	6.0	22.75	*p* < 0.001*** B > C,D, A
	In a relationship - B	59	24.82	3.27	26.0	16	28	22.5	27.00	
	Widowed - C	31	20.71	5.64	21.0	8	28	18.0	25.50	
	Divorced - D	9	18.22	6.96	20.0	3	27	16.0	22.00	
Professional activity	Mental work - A	18	26.28	2.63	27.0	16	28	26.25	27	*p* = 0.001*** A > B,C
	Physical work - B	17	23.00	3.97	23.0	15	28	21.00	26	
	Not occupationally active - C	70	21.44	6.23	22.5	3	28	19.00	27	
Material situation	Very good - A	16	27.00	0.63	27.0	26	28	27	27	*p* < 0.001*** A > B > C
	Good - B	59	24.21	3.50	26.0	13	28	22	27	
	Average or bad - C	30	16.80	6.44	18.5	3	27	14	21	
Inhabitation	Alone	22	17.18	7.06	17	3	28	14	22	*p* < 0.001**
	With family	83	23.94	4.35	26	3	28	21	27	

*Note * * – Kruskal-Wallis test; ** p – Mann-Whitney test; *** p – Kruskal-Wallis test + post-hoc analysis (Dunn’s test)

*Abbreviations* SD, standard deviation; ACDS, Adherence in Chronic Diseases Scale; n, number of participants; Q1, lower quartile; Q3, upper quartile

In Table 5, the results of a univariate analysis of the impact of selected variables on the level of adherence to therapeutic recommendations by the surveyed patients are presented. The duration of the disease, the presence of diabetes, rhythm disorders, arterial hypertension, and atherosclerosis did not have a statistically significant impact on adherence to therapeutic recommendations by the respondents (*p* > 0.05). However, the presence of obesity and respiratory system diseases among the respondents was a statistically significant factor influencing the level of adherence (*p* < 0.05). Adherence was higher in individuals without obesity and respiratory diseases. Furthermore, the level of adherence was better with fewer hospitalizations experienced by the patients. A higher level of knowledge among the respondents was associated with a higher level of adherence to therapeutic recommendations (*p* < 0.05). Specifically, among the participants, 9 patients had a very bad or bad level of knowledge, 66 patients had a rather good level of knowledge, and 30 patients had a good or very good level of knowledge.

**Table 5:** The results of a univariate analysis of the influence of clinical variables on the level of adherence to therapeutic recommendations in the surveyed patients

Variable			N		ACDS [score]													p	
					Mean		SD		Median		Min		Max		Q1		Q3		
Disease duration	Less than 1year	14		21.71		8.27		24.5		3		28		21.25		27		0.416*	
	1–4 years	54		23.09		5.38		26.0		3		28		21.00		27			
	5 years or more	37		22.00		5.09		22.0		8		28		19.00		27			
Diabetes	No	62		22.32		6.14		24		3		28		21		27		0.867**	
	Yes	43		22.81		5.08		25		8		28		20		27			
Atherosclerosis	No	86		22.61		5.81		25		3		28		21.0		27		0.626**	
	Yes	19		22.11		5.38		23		10		28		19.5		27			
Obesity	No	81		23.02		5.53		26.0		3		28		20.00		27.00		0.044**	
	Yes	24		20.83		6.11		22.5		3		27		19.25		25.25			
Hypertension	No	42		21.71		6.75		23.5		3		28		20.25		26		0.324**	
	Yes	63		23.06		4.89		25.0		8		28		20.00		27			
Respiratory diseases	No	83		23.11		5.57		26.0		3		28		21.00		27.00		0.005**	
	Yes	22		20.30		5.80		20.5		3		27		17.25		25.92			
Cardiac rhythm disorders	No	73		22.99		5.59		26		3		28		21.00		27		0.175**	
	Yes	32		21.46		5.94		22		3		28		19.25		27			
Hospitalizations for exacerbation of HF in the last year	Lack of hospitalizations - A	47		24.38		5.74		27.0		3		28		24.00		27.00		< 0.001 *** A > B > C	
	1–2 hospitalizations - B	46		21.58		5.57		22.5		3		28		19.25		26.00			
	More than 2 hospitalizations - C	12		18.83		3.24		19.0		14		24		16.00		21.25			
Level of knowledge	Very bad or bad - A	9		11.56		8.26		10		3		24		3.00		16		< 0.001*** C > B > A	
	Rather good - B	66		22.07		4.23		22		13		28		20.00		26			
	Good or very good - C	30		26.80		1.13		27		22		28		26.25		27			

*Notes * * – Kruskal-Wallis test; ** p – Mann-Whitney test; *** p – Kruskal-Wallis test + post-hoc analysis (Dunn’s test)

*Abbreviations* SD, standard deviation; ACDS, Adherence in Chronic Diseases Scale; n, number of participants; Q1, lower quartile; Q3, upper quartile

The multivariable linear regression model showed that significant (*p* < 0.05) independent predictors of ACDS score are: (1) being in a relationship – the regression coefficient is 5.318, indicating that it increases the ACDS score by an average of 5.318 points compared to being single, (2) widowhood – the regression coefficient is 5.473, indicating that it increases the ACDS score by an average of 5.473 points compared to being single, (3) average or poor financial situation – the regression coefficient is -9.353, indicating that it decreases the ACDS score by an average of 9.353 points compared to a very good financial situation, (4) obesity – the regression coefficient is -2.684, indicating that it decreases the ACDS score by an average of 2.684 points, and (5) respiratory system diseases – the regression coefficient is -2.429, indicating that they decrease the ACDS score by an average of 2.429 points **(**Table 6**)**.

**Table 6:** The results of the multivariable regression analysis of the impact of sociodemographic and clinical variables on the level of adherence to therapeutic recommendations by the surveyed patients

Variable		Parameter	95%CI		*p*
Education	Elementary or gymnasium	ref.			
	Vocational	0.488	-2.781	3.757	0.77
	Secondary school	2.821	-0.529	6.17	0.102
	Higher education	2.286	-2.413	6.984	0.343
Marital status	Single	ref.			
	In a relationship	5.318	0.859	9.777	0.022 *
	Widowed	5.473	1.142	9.804	0.015 *
	Divorced	4.385	-0.105	8.875	0.059
Professional activity	Mental work	ref.			
	Physical work	2.548	-1.925	7.021	0.267
	Not occupationally active	1.531	-2.667	5.73	0.477
Material situation	Very good	ref.			
	Good	-4.169	-8.796	0.458	0.081
	Average or bad	-9.353	-14.53	-4.177	0.001 *
Inhabitation	Alone	ref.			
	With family	2.207	-0.486	4.9	0.112
Obesity	No	ref.			
	Yes	-2.684	-4.515	-0.853	0.005 *
Respiratory diseases	No	ref.			
	Yes	-2.429	-4.385	-0.472	0.017 *
Disease duration	Less than 1year	ref.			
	1–4 years	-0.163	-3.014	2.688	0.911
	5 years or more	0.672	-2.557	3.902	0.684

*Notes * * – statistically significant relationship (*p* < 0.05); p – multiple linear regression

## Discussion

HF is considered one of the lifestyle diseases, with the number of patients affected steadily increasing both in Poland and worldwide. This has led to growing interest in the issue of non-adherence to therapeutic recommendations among chronically ill patients. In many cases, adherence is influenced by sociodemographic characteristics, medical aspects, and health behaviors [12–17]. These encompass conscientiously following doctor’s recommendations, maintaining a balanced diet, engaging in prescribed exercises, and avoiding harmful substances. Adherence to therapeutic recommendations and consistent medication intake are crucial elements in the recovery process and improving patients’ HRQoL.

While similar studies have been conducted, our research uniquely focuses on the specific sociodemographic factors influencing adherence to therapeutic recommendations among heart failure patients in a Polish population. This study adds new insights by identifying distinct predictors of adherence, such as relationship status, type of work, and financial situation, which have not been extensively explored in this context. Additionally, our use of the ACDS provides a comprehensive assessment tool that enhances the understanding of adherence behaviors in chronic disease management.

The objective of our research was to assess the level of adherence to therapeutic recommendations among HF patients. The study involved 105 individuals – 39 women (37.14%) and 66 men (62.86%). Our findings indicate that 34.29% of respondents exhibited a high level of adherence, while 39.05% demonstrated moderate adherence, with only 26.67% of patients showing low adherence. This aligns with the results of a study by Jarrah et al. [26]. , similarly, 33.5% exhibited a high level of adherence. Similar results were obtained by Kubica et al. [24] where it was also observed that the largest group of patients adhered to recommendations at a moderate level. In their more recent study, Kubica et al. [27] achieved comparable results, where, according to the ACDS scale, the majority, as much as 44.8% of individuals, adhered to recommendations at a moderate level. Additionally, a large-scale Polish study reported a 6.9% adherence rate to therapeutic recommendations [28].

The analysis of our research results revealed a statistically significant correlation between adherence to therapeutic recommendations and specific sociodemographic factors and coexisting diseases among the studied patient group. Adherence was notably higher among individuals with higher education compared to those with secondary education, and it was significantly higher among those with secondary education than those with primary, lower-secondary, and vocational education. Higher education often correlates with increased awareness and a higher socioeconomic status, which can enhance adherence to therapeutic recommendations [14, 29]. Moreover, having greater financial resources was associated with better adherence [29]. Furthermore, adherence levels were notably higher among individuals in relationships, those engaged in mental work, and those with good financial standing. Our findings align with those of Jarrah et al. [26], whose study showed that HF patients who were in relationships, better educated, and had health insurance exhibited high adherence levels. Additionally, individuals in stable relationships and living with their families demonstrated high adherence, suggesting that health behaviors may be influenced by the patient’s immediate environment.

Clinical factors related to HF and its treatment significantly influence the level of adherence to recommendations by patients with HF. In our study, the adherence level was higher in individuals without obesity and those not suffering from respiratory diseases. There was also a significant relationship between patient hospitalizations and adherence to recommendations. The level of adherence was better the fewer hospitalizations the patients experienced. The results of our research are consistent with studies available in the literature [28].

An intriguing finding from our study was the absence of a correlation between gender and adherence level in HF patients. While there are limited publications exploring gender’s influence on HF adherence, those that do suggest a positive impact on male patients [30–32]. It raises the question of whether men genuinely adhere better to treatment or if this trend stems from the support and involvement of women in caring for their family members. Conversely, factors such as age, place of residence, and disease duration did not significantly impact adherence. Given the conflicting findings in the literature, there is a need to reevaluate these factors in future studies.

Upon reviewing studies by other authors, slight variations in results were observed. Garred et al. [13] in their analysis of adherence to therapeutic recommendations, found that advanced age was associated with poorer adherence and a higher frequency of discontinuing medication doses. Conversely, Czekierda and Chojnacka-Kowalewska [33], found no relationship between hospitalizations and health behavior outcomes. They also observed no significant correlation between rural or urban residency and health behavior outcomes. However, Wysokiński and Dmowska [34] reported better health behaviors among seniors living in urban areas, while gender differences were noted, with women achieving higher scores. Additionally, Szlenk-Czyczerska et al. [35] found that individuals with shorter illness durations more frequently engaged in health-promoting behaviors.

Patients’ understanding of their own disease significantly impacts the treatment process [14]. It enables the recognition of alarming symptoms and slows disease progression through adherence to proper health habits. Our study validated a statistically significant correlation between knowledge and adherence to therapeutic recommendations. This is consistent with research by Szlenk-Czyczerska et al. [35], where respondents with high knowledge levels exhibited better health-related behaviors. Similarly, Piejko et al. [36] noted significant dietary errors among respondents, suggesting inadequate knowledge or incorrect dietary habits. Additionally, Krzemińska et al. [37] found that patient education improved quality of life by increasing the frequency of proper health behaviors.

The findings from this study can inform modifications to care plans for HF patients. Understanding factors influencing patients’ health behaviors can tailor personalized therapeutic recommendations, enhancing adherence. Attention to patients’ disease knowledge and quality of life is crucial for improving adherence levels. Further research involving a larger patient cohort would deepen our understanding of this topic.

### Study limitations

While this study provides valuable insights into the factors impacting adherence to therapeutic recommendations among HF patients, it is important to recognize certain limitations. Firstly, relying on self-reported data introduces the possibility of recall bias, potentially affecting the accuracy of information regarding medical history, lifestyle habits, and adherence behavior. Additionally, the cross-sectional nature of the study limits our ability to establish causal relationships between variables. The relatively modest sample size may also restrict the generalizability of findings to larger populations of HF patients. Furthermore, the study primarily focuses on sociodemographic and clinical factors, omitting other potentially relevant variables such as psychological factors or medication side effects. A major limitation of this study is its single-center design, which limits the generalizability of the findings; therefore, future multicenter studies are needed to validate these results across diverse populations. The sample size of 105 participants in our study should be larger, but it considered adequate for conducting statistical analyses and drawing meaningful conclusions. Despite the relatively small sample size, our study adheres to rigorous methodological standards, and the findings contribute valuable insights into the factors influencing adherence to therapeutic recommendations among HF patients.

### Practical implications

The practical implications of this study underscore the importance of tailored interventions to improve adherence to therapeutic recommendations among HF patients. Healthcare providers should prioritize patient education, ensuring clear communication of treatment plans and addressing any misconceptions. Additionally, implementing socioeconomic support systems can assist patients facing financial difficulties, facilitating access to essential resources. Regular screening for comorbidities such as obesity and respiratory diseases is vital for early detection and intervention. Embracing a patient-centered approach, where treatment plans are individualized to patients’ preferences and lifestyles, can foster greater engagement and adherence. Behavioral interventions, including cognitive-behavioral therapy, offer promising avenues for overcoming adherence barriers and promoting healthier habits. Lastly, fostering collaboration among healthcare professionals ensures comprehensive care delivery, catering to the diverse needs of HF patients. By embracing these strategies, healthcare providers can optimize adherence and enhance patient outcomes in HF management.

### Future research

Addressing these limitations in future research could enhance our understanding of adherence behavior in HF patients. Longitudinal study designs could establish temporal relationships between variables and improve the reliability of findings. Objective measures of adherence, such as medication adherence monitoring devices, could provide more precise data. Investigating the impact of psychological factors, social support, and healthcare system-related factors on adherence could offer a more comprehensive perspective. Also, future research could incorporate cognitive assessment tools to explore the impact of cognitive function on treatment adherence more comprehensively, especially in older age groups where cognitive dysfunction may be more prevalent. Additionally, developing and evaluating interventions tailored to the specific needs of HF patients could optimize patient outcomes. Collaborating with multidisciplinary teams, including statisticians and behavioral scientists, could enhance the methodological rigor and comprehensiveness of future studies in this research area.

## Conclusions

The level of adherence to therapeutic recommendations among patients with HF is moderate. Higher education, being in a relationship, engaging in mental work, good financial situation, and living with family are significant sociodemographic factors influencing the level of adherence to therapeutic recommendations for patients with HF. Low adherence is observed in individuals with obesity, respiratory diseases, and patients frequently hospitalized due to exacerbations of HF. The knowledge of the respondents also significantly affects adherence to therapeutic recommendations for patients with HF. Also, several independent predictors that increase the ACDS score have been identified, including being in a relationship, widowhood, and having an average or poor financial situation. Conversely, factors such as obesity and respiratory diseases were associated with a decrease in the ACDS score.

## Data Availability

The datasets generated and/or analyzed during the present study are available from the corresponding author upon reasonable request.

## Abbreviations

ACDS: Adherence in Chronic Diseases Scale
ATLAS: Assessment of Treatment with Lisinopril and Survival
HF: Heart failure
HFA: Heart Failure Association
HFrEF: Heart failure with reduced ejection fraction
HRQoL: Health-related quality of life
